# Characterization and Immune Functions of LcβLectin from Large Yellow Croaker (*Larimichthys crocea*): A Potential Antiviral Defense Molecule

**DOI:** 10.3390/ijms26073251

**Published:** 2025-03-31

**Authors:** Jiawei Zhang, Hongling Wu, Ying Huang, Yao Yang, Dinaer Yekefenhazi, Wenzheng Zou, Fang Han

**Affiliations:** 1 State Key Laboratory of Mariculture Breeding, Fujian Provincial Key Laboratory of Marine Fishery Resources and Eco-Environment, Fisheries College, Jimei University, Xiamen 361000, China; z13954765720@163.com (J.Z.); 18878468726@163.com (H.W.); huangying02082022@163.com (Y.H.); yangyaoyj@163.com (Y.Y.); s2745005@ed.ac.uk (D.Y.); 2 Roslin Institute, Royal (Dick) School of Veterinary Studies, University of Edinburgh, Midlothian EH8 9YL, UK

**Keywords:** galectin, LcβLectin, *Larimichthys crocea*, large yellow croaker iridovirus (LYCIV), macrophage, antiviral defense

## Abstract

Large yellow croaker iridovirus (LYCIV) poses a significant threat to the large yellow croaker (*Larimichthys crocea*) aquaculture industry due to its rapid transmission and high lethality. Galectins, as evolutionarily conserved carbohydrate-binding lectins and pattern recognition receptors (PRRs) in the innate immune system, play crucial roles in immune responses. In this study, we characterized the beta-galactoside-binding lectin from large yellow croaker (*LcβLectin*) and explored its potential as a disease resistance gene against LYCIV. The full-length cDNA of *LcβLectin* was cloned and found to contain conserved elements, such as β-galactoside-binding motifs, HNPR, and WCEEHR domains. Using *L. crocea* head-kidney macrophages (LCM10), we demonstrated that recombinant LcβLectin significantly inhibits LYCIV-induced cytopathic effects and reduces macrophage apoptosis, highlighting its key role in viral defense. Moreover, the overexpression of *LcβLectin* in LCM10 cells followed by transcriptomic analysis revealed its substantial regulatory effects on key immune-related signaling pathways, including C-type lectin signaling, p53 signaling, and Toll-like receptor signaling pathways. Collectively, our findings suggest that LcβLectin enhances fish resistance to viral diseases by augmenting immune system function and activating immune-related pathways, providing valuable insights into the innate immune mechanisms of aquatic species and potential strategies for disease prevention in aquaculture.

## 1. Introduction

The innate immune system functions as the body’s primary defense mechanism against various pathogens, effectively resisting their invasion. In both vertebrates and invertebrates, pathogen recognition is achieved by pattern recognition receptors (PRRs), which identify pathogen-associated molecular patterns (PAMPs) located on the surface of pathogens [1]. This recognition process often involves protein–carbohydrate interactions, primarily facilitated by soluble and cell-associated lectins [2]. Lectins constitute a vital class of PRRs that selectively attach to carbohydrate molecules on the surfaces of pathogens. This binding enables the pathogens’ elimination through opsonization and phagocytosis, while also enhancing the respiratory burst response of immune cells. Moreover, the specific binding capacity of lectins not only supports intercellular interactions and signal transduction but also represents a fundamental mechanism by which organisms detect and respond to foreign pathogens [3]. Initially categorized into C- and S-type lectins (later identified as galectins) in 1988, animal lectins are characterized by unique sequence motifs and structural folds, with subsequent discoveries of P-, I-, L-, and F-type lectins, as well as pentraxins, ficolins, and calnexin [4].

Galectins constitute a family of evolutionarily conserved, soluble, Ca^2+^-independent lectins characterized by their affinity for β-galactosides. These proteins bind to polysaccharides on the surfaces of pathogenic bacteria, fungi, and parasites, indicating their role as PRRs in the innate immune system [5]. Galectins are broadly classified into three types based on their structural variations: prototype (Galectin-1, -2, -5, -7, -10, -11, -13, -14, -15), chimaera-type (Galectin-3), and tandem-repeat (Galectin-4, -6, -8, -9, -12) [6]. Notably, galectins, a highly pleiotropic family of proteins, mediate multiple signaling pathways and play pivotal roles in diverse biological processes, including inflammation, immune defense, and muscle development [7,8,9]. Importantly, galectins are central to the innate immune response of the host, facilitating processes that resist and eliminate pathogen invasion [9]. They are recognized as significant PRRs with essential functions in innate immunity [10].

Beta-galactoside-binding lectin is a member of the galectin family, characterized by a carbohydrate recognition domain (CRD) and a distinct β-galactoside binding motif. Additionally, the glycobiology of lectins, including their recognition of specific carbohydrate structures, has been extensively reviewed by Gabius et al. [11,12]. However, it has not yet been classified within the galectin family of the large yellow croaker. Notably, this lectin exhibits a higher sequence homology with Galectin-1, which belongs to the prototype galectin subfamily in other fish species. The functional implications of galectin-1 have been extensively studied, revealing its role in the immune defense mechanisms of fish. For instance, Drgal1-L2 in zebrafish (*Danio rerio*) has been shown to interact with the galactose β1, 4-N-acetylglucosamine (LacNAc) present on the envelope of the infectious hematopoietic necrosis virus (IHNV). This interaction facilitates the recognition and binding of Drgal1-L2 to the virus’s glycosylated envelope, thereby disrupting its adhesion to host cells. Moreover, the pre-incubation of IHNV with recombinant Drgal1-L2 resulted in a greater than 40% reduction in the binding rate of IHNV to the epithelial cell line (EPC) [13]. In head kidney leukocytes from European sea bass (*Dicentrarchus labrax*), the addition of rSbGal-1 produced a dose-dependent decrease in the respiratory burst of leukocytes. In contrast to fish infected solely with the sea bass nervous necrosis virus (SBNNV), a significant reduction in the expression levels of the cytokines IL-1, TNF-α, and Mx was observed in the brains of those that received simultaneous injections of the virus and rSbGal-1 [14]. Therefore, we speculate that beta-galactoside-binding lectin may possess potential antiviral properties.

Aquaculture represents the fastest growing sector in global food production and has become an essential component of the global food supply [15]. However, its sustainable development encounters significant challenges, primarily stemming from disease outbreaks [16]. Fish diseases are diverse and highly transmissible, with global aquaculture losses estimated to surpass USD 6 billion annually [17]. Therefore, addressing disease challenges and minimizing losses are critically important for the entire aquaculture industry. The large yellow croaker (*Larimichthys crocea*) is the most extensively cultivated mariculture fish in China. However, high-density net-cage farming often causes outbreaks of various diseases, with large yellow croaker iridovirus (LYCIV) being the most prevalent and devastating, leading to enormous annual economic losses [18,19]. Infected large yellow croakers exhibit clinical symptoms, including pale gills, hepatomegaly, and necrosis of the spleen and kidney [20]. Given the frequent occurrence and severe harm of LYCIV infection, it is essential to explore the mechanisms underlying resistance mechanisms in the large yellow croaker to iridovirus infection.

In this study, the beta-galactoside-binding lectin gene derived from the large yellow croaker (*LcβLectin*) was identified as a key candidate gene for resistance to LYCIY. The gene was successfully cloned and underwent comprehensive functional characterization. We analyzed the role of the LcβLectin protein in enhancing the resistance of large yellow croaker macrophages against iridovirus infection using flow cytometry. Then, *LcβLectin* was overexpressed and underwent combined transcriptome sequencing analysis to explore its immune regulatory mechanisms during LYCIV infection. These findings reveal the essential role of LcβLectin as a key pattern recognition receptor (PRR) and provide novel insights into the host defense mechanism, thereby advancing our understanding of innate immune responses in marine fish species.

## 2. Results

### 2.1. Sequence Analysis and Molecular Characteristics of LcβLectin

**Figure 1:**
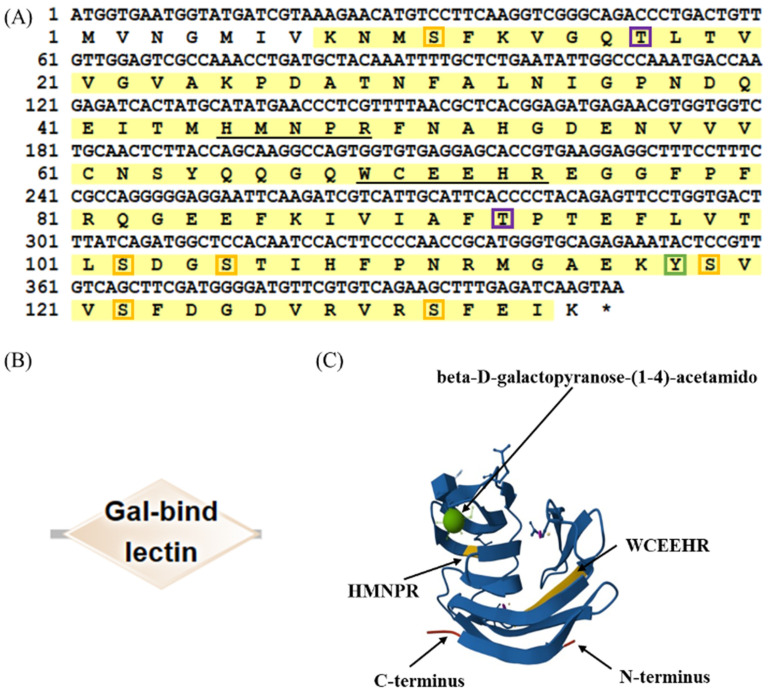
Sequence characterization and protein structure analysis of LcβLectin. (**A**) The coding and deduced amino acid sequence of LcβLectin. Carbohydrate recognition domains (CRDs) are highlighted in yellow. The characteristic β-galactoside-binding motifs, H-NPR and W-EE-, are underlined. Serine phosphorylation sites are indicated by orange boxes, threonine phosphorylation sites by purple boxes, and tyrosine phosphorylation sites by green boxes; * means the stop code. (**B**) The conserved domain and (**C**) the tertiary structure of the LcβLectin protein.

The full-length cDNA of *LcβLectin* is 843 base pairs (bp), encompassing an open reading frame (ORF) of 409 bp that encodes 136 amino acids. The predicted molecular weight is 15.17 kDa, and the theoretical isoelectric point (pI) is 5.50. LcβLectin contains nine phosphorylation sites, including six serine (Ser) phosphorylation sites, two threonine (Thr) phosphorylation sites, and one tyrosine (Tyr) phosphorylation site (Figure 1A). The predictions indicated that the LcβLectin protein lacks signal peptides and transmembrane domains (Figure 1B). Furthermore, SMART protein domain prediction revealed that LcβLectin contains a carbohydrate recognition domain (CRD), which includes two highly conserved β-galactoside-binding motifs, H-NPR and WCEEHR (Figure 1C).

**Figure 2:**
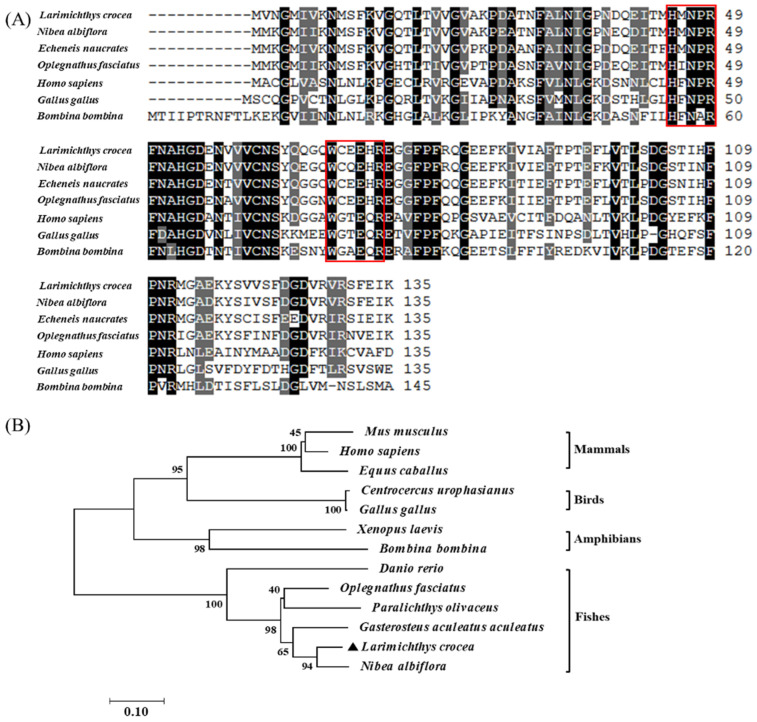
Sequence alignment and phylogenetic analysis of LcβLectin and its homologs in other species. (**A**) Multiple sequence alignment showing high conservation of key amino acids (black shadows) and sugar-binding motifs (red boxes, H-NPR and W-EE-). These motifs are critical for β-galactoside binding, confirming LcβLectin’s classification as a galectin. (**B**) Phylogenetic analysis demonstrates that LcβLectin clusters closely with galectins from other fish species, particularly yellow drum (*Nibea albiflora*), indicating strong evolutionary conservation. Black triangle marks the study subject—large yellow croaker. The numbers at the nodes represent bootstrap confidence values from 1000 replicates (%), and the scale bar (0.10) indicates genetic distance. GenBank accession numbers for sequences used are listed in Table 1.

Amino acid alignment revealed the presence of both sugar-binding motifs (H-NPR and W--EE-), exhibiting 90.37% and 84.44% sequence identity to yellow drum (*Nibea albiflora*) and live sharksucker (*Echeneis naucrates*), respectively. Additionally, the phylogenetic tree analysis demonstrated that LcβLectin clusters with galectins from other fish species, showing the closest evolutionary relationship with yellow drum (*N. albiflora*). In contrast, mammals, birds, and amphibians form a separate cluster, consistent with their taxonomic divergence [21] (Figure 2, Table 1).

### 2.2. Tissue Expression Pattern and Cellular Distribution

**Figure 3:**
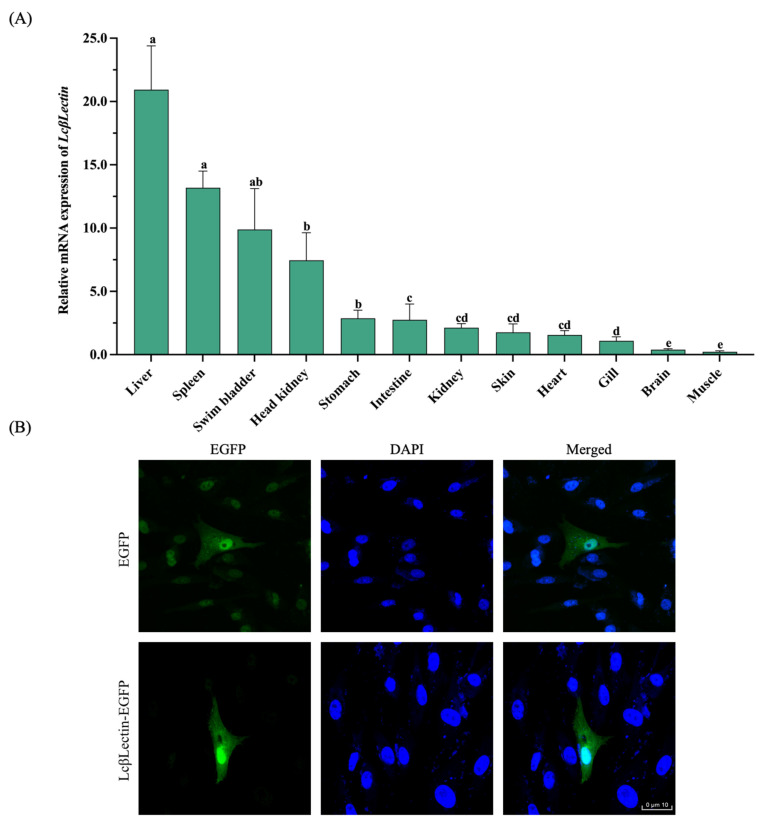
Tissue expression profile and subcellular localization of LcβLectin. (**A**) Tissue expression patten of *LcβLectin* in the large yellow croaker. *β-actin* was used as an internal control. Values represent mean ± SD of three biological replicates. The lowercase letters above the error bars in indicate statistically significant differences between tissue groups based on one-way ANOVA followed by Duncan’s multiple range test (*p* < 0.05). (**B**) Subcellular localization of LcβLectin in transfected LCM10 cells. The scale bar is 10 μm.

*LcβLectin* was expressed in all tested tissues, including kidney, spleen, liver, gills, head kidney (a specific organ in fish), skin, brain, stomach, muscle, heart, swim bladder, and intestine. Among these, the highest expression level was observed in the liver, followed by the spleen, swim bladder, head kidney, stomach, and intestine, while the lowest expression level was detected in muscles (Figure 3A). The subcellular localization results indicate that the LcβLectin protein is present in both the nucleus and cytoplasm of LCM10 cells (Figure 3B).

**Figure 4:**
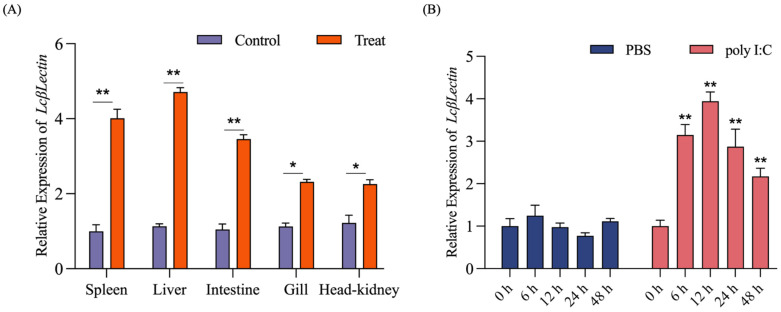
Induced expression of *LcβLectin*. (**A**) The effect of LYCIV infection on the expression of *LcβLectin*. (**B**) Temporal expression profile of *LcβLectin* in LCM cells after poly I:C challenge. Data are mean ± SD (n =3). Statistical significance was determined by a one-way ANOVA followed by Duncan’s multiple range test. The symbols * and ** indicate significance levels of *p* < 0.05 and *p* < 0.01, respectively.

Following infection with LYCIV, a significant upregulation of *LcβLectin* expression was observed in the immune-related tissues of the large yellow croaker, including spleen, liver, intestine, gills, and head kidney. To further validate the response of *LcβLectin* to pathogens, LCM10 cells were stimulated with pathogen mimetics poly I: C. The results indicate that *LcβLectin* expression was significantly upregulated following stress (Figure 4).

### 2.3. Prokaryotic Expression and Purification of LcβLectin Protein

**Figure 5:**
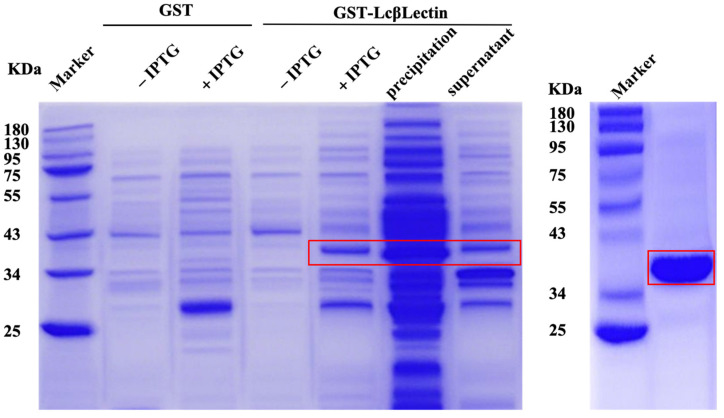
Prokaryotic expression and purification of GST-LcβLectin protein. The left figure shows the induced expression of the protein, and the right figure shows the protein purification. The red box indicates the GST-LcβLectin fusion protein.

The BL21 (DE3) strains containing pEGX-6p-1 and GST-LcβLectin were successfully expressed in the prokaryotic system. The results of the protein purification are shown in Figure 5. The size of the recombinant GST-LcβLectin protein was found to match the expected size of 41 kDa.

### 2.4. Neutralizing Virus Activity of Recombinant LcβLectin

**Figure 6:**
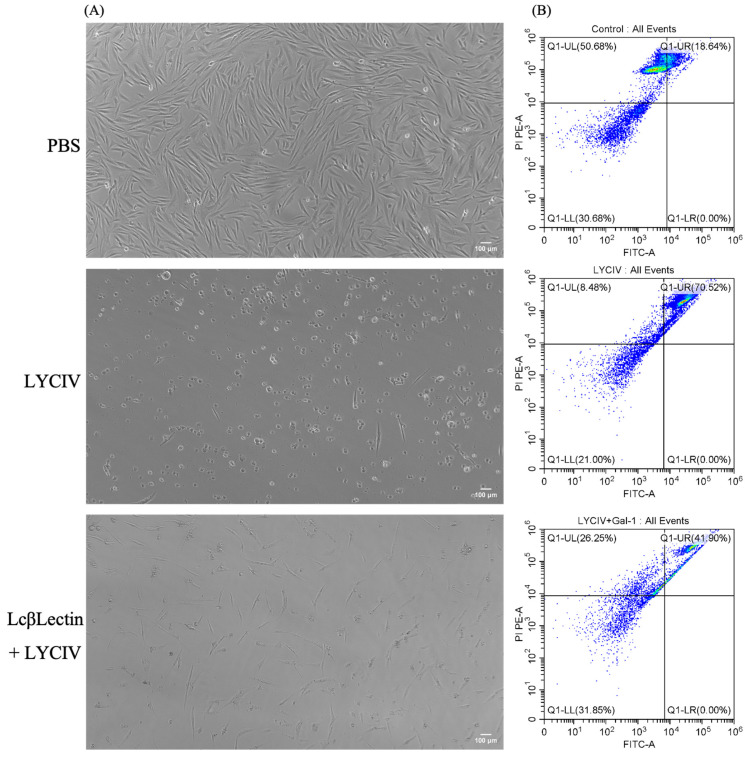
Neutralizing virus activity analysis of recombinant LcβLectin protein. (**A**) Morphological assessment of large yellow croaker macrophages under control (unstimulated) and virus-stimulated conditions. (**B**) Flow cytometry quantification of apoptosis rates in LCM10 cells following viral stimulation. Each group was assayed in three technical replicates, with a scale bar set to 100 μm.

The apoptosis of LYCIV-infected cells was assessed using flow cytometry to evaluate the neutralizing effect of the LcβLectin protein against the virus. The results show that cells cultured in media containing 2% BCS, PBS, or LcβLectin protein were not adversely affected. However, upon exposure to the LYCIV, the cells exhibited distinct cytopathic effect (CPE). Unlike normally growing monolayer cells, the morphology of the virus-stimulated cells became rounded or showed signs of perforation and rupture, leading to reduced adherence and causing them to float on the surface of the culture medium. In the experimental group where cells were co-incubated with LcβLectin protein and the LYCIV, cell viability was notably improved compared to cells subjected solely to the virus, demonstrating that LcβLectin mitigated the viral toxicity, as illustrated in Figure 6A. The flow cytometric analysis of apoptotic cells in all three groups yielded results consistent with those observed microscopically, as shown in Figure 6B. The highest apoptotic cell count was observed in the virus-stimulated group, reaching 70.52%. In contrast, the experimental group co-incubated with LcβLectin exhibited an apoptotic cell count of 41.90%, while the control group had a count of 18.64%.

### 2.5. Transcriptome Data Analysis and Identification of DEGs

Six libraries, labeled as c-1, c-2, c-3, LcLec-1, LcLec-2, and LcLec-3, were constructed from the total RNA extracted from the LCM10 cells. On average, each library yielded 7.90 Gb of clean bases and 52.50 M clean reads, with a Q30 quality score exceeding 96.98%. Approximately 89.41% to 89.84% of clean reads were successfully mapped to the *L. crocea* reference genome (NCBI GCA_003845795.1) (Table 2).

**Figure 7:**
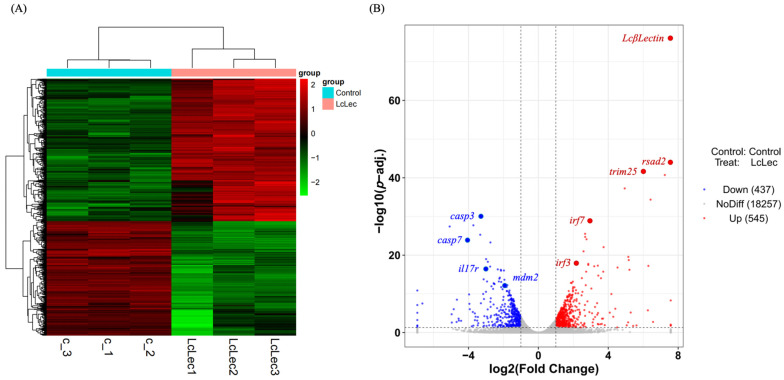
Analysis of transcriptome data and differentially expressed genes (DEGs). (**A**) Cluster analysis of RNA-Seq datasets using all DEGs (*p*-adj. < 0.05, log_2_|(Fold Change)| ≥ 1). The x-axis represents genes, with each column corresponding to an individual sample. Red indicates genes with high expression, while green represents genes with low expression. (**B**) Volcano plot illustrating DEGs in overexpressed samples. The two vertical dashed lines represent the twofold change threshold, and the horizontal dashed line denotes the *p*-adj = 0.05 significant threshold. Red dots indicate upregulated genes in this group, blue dots represent downregulated genes, and gray dots signify genes without significant differential expression.

Significant differences were observed between the control and experimental groups, accompanied by strong correlations among the three biological replicates (Figure 7A). A total of 982 DEGs were identified, comprising 545 upregulated and 437 downregulated genes. The differential expression is visually depicted in a volcano plot (Figure 7B).

### 2.6. Enrichment Analysis of DEGs

**Figure 8:**
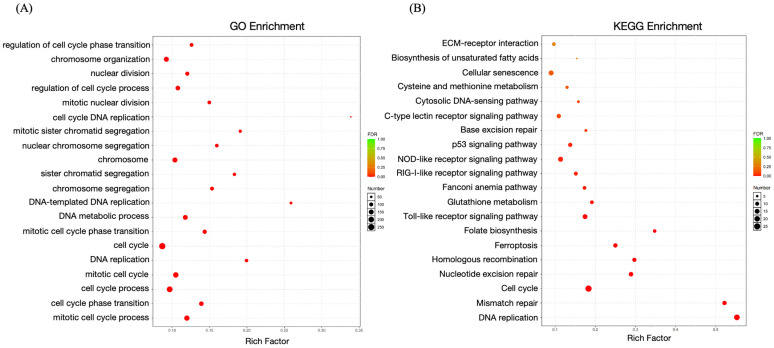
Functional enrichment analysis of DEGs after *LcβLectin* overexpression. (**A**) GO enrichment analysis highlights DEG involvement in key biological processes. (**B**) KEGG pathway enrichment identifies immune-related pathways. The x-axis “Rich Factor” indicates the proportion of DEGs enriched in each pathway relative to the total annotated DEGs. Dot size and color denote the number of DEGs and statistical significance (*p* < 0.05), respectively.

The GO enrichment analysis identified key biological processes, such as cellular and metabolic processes, that were significantly overrepresented. Furthermore, the KEGG pathway enrichment analysis shed light on numerous immune-associated signaling pathways (Figure 8). Specifically, twelve DEGs were significantly enriched in the C-type lectin receptor signaling pathway, eleven in the p53 signaling pathway, and fifteen in the Toll-like receptor signaling pathway. These findings underscore the immunological significance of the identified DEGs and their roles in essential cellular processes.

### 2.7. RT-qPCR Verification of DEGs

**Figure 9:**
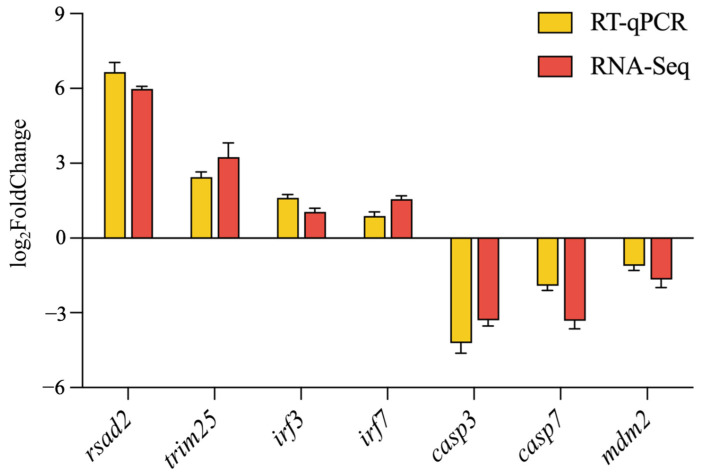
Comparative verification of the expression profiles of RNA-Seq to RT-qPCR.

To validate the accuracy and reliability of the DEGs, the expression levels of seven immune-related DEGs—four upregulated (*rsad2*, *trim25*, *irf3*, and *irf7*) and three downregulated (*casp3*, *casp7*, and *mdm2*)—were quantified. The observed expression patterns of these selected genes were consistent with the RNA-Seq data, thereby confirming the reliability of the RNA-Seq results (Figure 9).

## 3. Discussion

Non-specific immunity plays a crucial role in sustaining dynamic equilibrium, thwarting microbial infiltration, eliminating pathogens, and facilitating the development of adaptive immune responses. Galectin, a family of widely occurring sugar-binding proteins, are found across both invertebrate and vertebrate species and serve as integral components of the innate immune system. Acting as key mediators in cellular signaling, galectins are essential for various immune cell interactions and participate in fundamental biological processes, such as cell adhesion, immune response, antimicrobial activity, regulation of cell growth, and apoptosis. By binding to glycoproteins and glycolipids on microbial surfaces, galectins are recognized as a primary family of pattern recognition receptors (PRRs) that initiate and mediate innate immune responses. In this paper, we characterized the structure and biological role of a beta-galactoside-binding lectin, referred to as *LcβLectin*, in the large yellow croaker. LcβLectin contains a single CRD located at the C-terminus and possesses typical sugar-binding motifs. Only one Gal-binding lectin domain was found at the LcβLectin-conserved domain, and this feature is similar to that of chimera-type galectins [22]. Similar results were found in *Scylla paramamosain* [23] and *Eriocheir sinensis* [24]. The phylogenetic analysis based on protein sequence homology revealed that LcβLectin is highly conserved across mammals and teleost fish, showing significant similarity to the Galectin-1 of other bony fish species [25], including *N. albiflora* and *E. naucrates* [26].

Given that galectins directly interact with pathogens and modulate key components of both innate and adaptive immunity, they play a pivotal role in mediating host responses to infections through multiple mechanisms [6]. Nevertheless, research exploring the functions of galectins in viral infections is still comparatively scarce. In mammals, Gal-1 can bind to the glycan structures of influenza virus via its CRD, which inhibits viral hemagglutination and prevents viral entry into host cells [27]. Monocytes and macrophages primarily initiate immune responses by secreting factors, including IL-6, IL-8, IL-10, and macrophage migration inhibitory factor (MIF) [28,29,30]. The fGLec-1 of flounder *Paralichthys olivaceus* has shown effective antiviral properties, neutralizing lymphocystis disease virus (LCDV) virulence. Recombinant rOfGal-1 from black porgy (*Acanthopagrus schlegelii*) has been implicated in the defense against infections by iridovirus and viral hemorrhagic septicemia virus (VHSV) [31]. In Nile tilapia (*Oreochromis niloticus*), the incubation of monocytes/macrophages with recombinant rOnGal-3 protein significantly enhanced the transcription levels of IL-6, IL-8, IL-10, and MIF. Flow cytometry revealed that rOnGal-3-treated macrophages display increased bacterial phagocytosis, alongside a heightened respiratory burst response [32]. In our study, recombinant LcβLectin protein demonstrated antiviral activity within an LCM cell line, effectively delaying cytopathic effects (CPE) induced by LYCIV. The flow cytometry analysis further showed that LcβLectin mitigates LYCIV-induced cell apoptosis, indicating its capacity to neutralize LYCIV virulence. As a pattern recognition receptor, galectin activates macrophage phagocytosis upon binding to PAMPs [33].

The innate immune system identifies microbial presence and initiates mechanisms to eliminate potentially infectious threats. This detection relies on PRRs, which are germline-encoded receptors that monitor extracellular and intracellular environments for conserved microbial markers indicative of infection [34]. PRRs recognize PAMPs, serving as an effective alert system that triggers innate immune responses. Through PRRs, the immune system can identify various classes of pathogens based on their shared, conserved molecular patterns [35]. Interestingly, the overexpression of *LcβLectin* enriched the Toll-like receptor (TLRs) and C-type lectin receptor (CLRs) signaling pathways, both members of the PPRs family [34]. Similarly, in human synovial fibroblasts, galectin-3 has been shown to suppress inflammation by modulating IL-6 secretion induced by Toll-like receptor [36]. Additionally, Gal-3 has been found to promote inflammation and apoptosis by enhancing oxidative stress mediated by TLR-4 signaling in TC28a2 human chondrocyte cells [37]. In a mouse model, Gal-9 has been reported to inhibit TLR-mediated autoimmunity [38]. Overall, galectin proteins contribute to host immune defense by regulating various signaling pathways, highlighting their multifunctional involvement in immune modulation.

The overexpression of *LcβLectin* markedly upregulated the expression of *rsad2*, *trim25*, *irf3*, and *irf7*. *Rsad2* (also known as *viperin*), a highly conserved interferon-stimulated gene (ISG), plays an essential role in vertebrate antiviral immunity [39]. Typically, induced by IFN1 signaling, *rsad2* expression is highly elevated in response to interferon activation [40]. Studies also indicate that *rsad2* can be regulated by IRF1 and IRF3 independently of interferon. During infections by chikungunya virus (CHIKV) and reovirus, mitochondrial antiviral signaling protein (MAVS) activates IRF3, leading to *rsad2* upregulation, expanding its function beyond interferon-dependent pathways [41]. The upregulation of *rsad2* suggests that *LcβLectin* activates a broad-spectrum antiviral defense in host cells, limiting viral replication and preventing the spread of infection. Similarly, *trim25* plays a crucial role as a regulator of the RIG-I signaling pathway, mainly by promoting the K63-linked ubiquitination of RIG-I, which subsequently activates downstream interferon responses. Interferons are central to antiviral immunity, initiating the expression of various antiviral genes to enhance the host cell’s infection resistance [42]. The upregulation of *trim25* indicates that large yellow croaker cells engage the RIG-I pathway in response to iridovirus infection, likely related to *LcβLectin*’s role in early viral recognition. Furthermore, *irf3* and *irf7* are essential transcription factors within the interferon regulatory network, each playing distinct but complementary roles in the interferon signaling pathway. *Irf3* is rapidly activated during the early stages of viral infection, while *irf7* primarily amplifies the interferon response, sustaining the immune reaction [43]. In LCM10 cells, the upregulation of *irf3* and *irf7* may represent crucial steps in viral defense, indicating that *LcβLectin* not only initiates immune responses but also supports their maintenance and intensification to counter sustained viral pressure.

Acute inflammatory response is regarded as a vital component of the innate immune defense mechanism. The overexpression of *LcβLectin* can inhibit inflammation by decreasing the expression of *casp3*, *casp7*, and *mdm2*. Caspases were involved in immune response and apoptosis during bacterial or viral infections in teleost [44]. *Casp3* and *casp7* are key executors within the apoptosis pathway. Their downregulation may reduce premature cell apoptosis during viral infection, allowing host cells more time to combat infection. This extended cell survival benefits antiviral defense by exposing the virus to immune system responses for a longer period, enabling other antiviral mechanisms to ultimately clear infected cells. Furthermore, this downregulation may limit excessive virus-induced cell death, protecting tissues from damage associated with viral invasion [45]. Moreover, *mdm2*, a negative regulator of the p53 pathway, is downregulated, which may activate the p53 pathway. p53 plays multiple roles in cellular stress responses, including DNA repair and cell cycle arrest. Research has shown that p53 also enhances antiviral immunity by regulating a set of antiviral genes, including cytokines and interferon-related genes [46]. Thus, the downregulation of *mdm2* may further strengthen the immune defenses of large yellow croaker cells, providing multiple layers of protection against viral replication.

## 4. Materials and Methods

### 4.1. Sample Collection, RNA Extraction, and cDNA Synthesization

Large yellow croakers weighing 28.0 ± 8.5 g were sourced from Ningde, Fujian Province, China. Tissue samples including liver, spleen, swim bladder, head kidney, stomach, intestine, kidney, skin, heart, gill, brain, and muscle, were collected from three individuals and promptly stored at −80 °C. Total RNA was extracted from the above collected tissues using the TriZol total RNA isolation reagent (LABLEAD, Beijing, China), and cDNA was synthesized using All-in-One First-Strand Synthesis MasterMix (LABLEAD, China).

### 4.2. Cell Culture

The large yellow croaker head kidney macrophage (LCM10) cell line was kindly provided by Professor Qinghui Ai from Ocean University of China [47]. The cells were cultured at 28 °C in Dulbecco’s modified Eagle’s medium /F12 (DMEM/F12) medium supplemented with 15% FBS and 100 IU/mL penicillin–streptomycin. All the present experiments were approved by the Animal Care and Use Committee of Fisheries College at Jimei University.

### 4.3. Bioinformatics Analysis and Plasmid Construction

The open reading frame (ORF) sequences of *LcβLectin* (GenBank XM_010737784.3) were cloned into the pEGX-6p-1, pEGFP-N1, and pcDNA3.1(−)/myc-His B plasmids. The specific primers used are listed in Supplementary Table S1. Bioinformatics analysis were conducted following the protocol previously outlined by Huang [48]. The sequence alignment and domain prediction of LcβLectin amino acid (GenBank XP_010736086.1) were performed using ClustalOmega (http://www.clustal.org/omega/, accessed on 11 December 2024) and ExPASY (https://www.expasy.org, accessed on 11 December 2024). Signal peptide prediction was carried out using the SignalP server (https://services.healthtech.dtu.dk/services/SignalP-5.0/, accessed on 15 December 2024), while tertiary structure modeling was performed using the Phyre2 (https://www.sbg.bio.ic.ac.uk/phyre2/html/page.cgi?id=help, accessed on 20 December 2024), and visualization was facilitated by VMD 1.9.4 (https://www.ks.uiuc.edu/Research/vmd/vmd-new/devel.html, accessed on 20 December 2024). Furthermore, evolutionary relationships were examined by constructing a phylogenetic tree using MEGA11, applying the Neighbor-Joining algorithm, and incorporating sequences from multiple species.

### 4.4. Real-Time Quantitative PCR (RT-qPCR)

The expression profiles of the *LcβLectin* gene were evaluated across twelve different tissues, including liver, spleen, swim bladder, head kidney, stomach, intestine, kidney, skin, heart, gill, brain, and muscle. Furthermore, alterations in gene expression following LYCIV infection were assessed in immune-related tissues including intestine, liver, gills, head kidney, and spleen.

The response to poly I:C stimulation was specifically examined in *L. crocea* head kidney macrophage (LCM10). Cells were seeded in 6-well plates (1 × 10^6^ cells/well) and allowed to adhere for 24 h. The medium was replaced with fresh medium containing poly I:C (10 μg/mL). Cells were harvested at 0, 6, 12, 24, and 48 h post-stimulation for RNA extraction and RT-qPCR analysis. *β-actin* was served as the internal reference gene. Relative gene expression levels were quantified using the 2^−ΔΔCT^ method [49]. RT-qPCR was conducted using Taq SYBR^®^ Green qPCR Premix (LABLEAD Biotech, China).

### 4.5. LcβLectin Subcellular Localization

To evaluate the subcellular localization of LcβLectin, the recombinant expression plasmid LcβLectin-pEGFP-N1 (LcβLectin-EGFP) was transfected into LCM10 cells via transient transfection using an electro-transfer apparatus, with the empty vector pEGFP-N1 as control. After a 24 h transfection at 28 °C, the cells were fixed with 4% paraformaldehyde (LABLEAD, China), permeabilized for 10 min, and stained with DAPI. The localization of LcβLectin-EGFP was observed using fluorescence microscopy (Leica SP8, Wetzlar, Germany).

### 4.6. Prokaryotic Expression and Purification of LcβLectin

The expression plasmid LcβLectin-pEGX-6p-1 (GST- LcβLectin) was constructed to obtain recombinant LcβLectin (rLcβLectin) protein. Protein expression was induced by adding isopropyl β-D-1-thiogalactopyranoside (IPTG) to a final concentration of 0.05 mM, followed by incubation at 20 °C for 16 h. Following induction, the (r)LcβLectin were purified using glutathione S-transferase (GST) affinity chromatography, according to the protocol provided by BeyoGold™ GST-tag Purification Resin (Beyotime, Shanghai, China).

### 4.7. Annexin V-FITC/PI Flow Cytometer Assay

LCM10 cells were co-incubated with (r)LcβLectin and an iridovirus-containing suspension to monitor ongoing cytopathic effects (CPEs) and simultaneously measure the apoptotic rate of the infected cells via flow cytometry. Cells (1 × 10^7^ CFU/mL) were seeded into a 6-well plate and cultured for 24 h. After removing the culture medium, each well was treated with 480 μL of iridovirus dilution and 480 μL of PBS buffer, followed by treatment with 480 μL of iridovirus dilution in combination with 200 μg/mL of (r)LcβLectin. The control group received an equivalent volume of PBS buffer. Following a 3 h incubation, the supernatant was removed, and 1 mL of culture medium containing 2% bovine calf serum (BCS) was added to each well, followed by further incubation with continuous monitoring cell conditions. Each treatment group was performed in triplicate. Cells were processed according to the Annexin V-FITC/PI apoptosis kit instructions (LABLEAD, China).

### 4.8. LcβLectin Overexpression

LCM10 cells were transfected with the myc-LcβLectin recombinant plasmid and the pcDNA3.1(−)/myc-His B empty vector (control) via transient transfection using an electro-transfer apparatus (5 μg plasmid per 1 × 10^6^ cells). Twenty-four hours after transfection, cell samples from three replicates were collected for RNA extraction using the protocol provided by the TransZol Up Plus RNA Kit (TransGen, Beijing, China).

### 4.9. Library Construction, Sequencing, and Transcriptomic Analysis

Total RNA was extracted from cells transfected with pcDNA3.1 and myc-LcβLectin recombinant plasmid. The qualified RNA samples were subsequently utilized for library construction. Paired-end (PE) sequencing was performed on the Illumina NovaSeq 6000 platform by Shanghai Bioprofile Technology Co., Ltd. (Shanghai, China) Transcriptomic analysis was conducted using the reference genome of *L. crocea* (NCBI GCA_003845795.1). Differential expression analysis was performed using the DESeq2 package (https://support.bioconductor.org/tag/DeSeq2/, accessed on 5 January 2025), identifying significantly differentially expressed genes (DEGs) based on the criteria of adjusted *p*-value (*p*-adj.) < 0.05 and log_2_|(Fold Change)| ≥ 1.

DEGs were annotated and subjected to functional enrichment analysis using the clusterProfiler R package (v4.0). GO terms and KEGG pathways were considered significantly enriched if *p*-adj. < 0.05. The reference GO and KEGG databases were retrieved from the Gene Ontology Consortium (http://geneontology.org, accessed on 5 January 2025) and KEGG PATHWAY Database (https://www.genome.jp/kegg/pathway.html, accessed on 5 January 2025), respectively. The visualization of enriched terms was generated using the ggplot2 package (https://ggplot2.tidyverse.org, accessed on 5 January 2025).

### 4.10. RT-qPCR Verification

To validate the transcriptome sequencing results, seven immune-related differentially expressed genes—including radical S-adenosyl methionine domain-containing 2 (*rsad2*), tripartite motif 25 (*trim25*), interferon regulatory factor 3 (*irf3*), interferon regulatory factor 7 (*irf7*), caspase-3 (*casp3*), caspase-7 (*casp7*), and murine double minute 2 (*mdm2*)—were selected for RT-qPCR, with *β-actin* serving as the internal reference gene. The specific primes are shown in Table 1.

### 4.11. Statistical Analysis

All statistical analyses were performed with SPSS 26.0 (IBM, Armonk, NY, USA). Experimental data were presented as the mean ± standard error of the mean (SEM). A one-way ANOVA followed by Duncan’s multiple range test was employed to assess the statistical data. Differences were considered statistically significant when *p* < 0.05.

## 5. Conclusions

In conclusion, our study has comprehensively characterized the beta-galactoside-binding lectin (*LcβLectin*) from the large yellow croaker, revealing its conserved structural motifs and immune functions. We found that (r)LcβLectin inhibits the cytopathic effects of large yellow croaker iridovirus (LYCIV) and reduces macrophage apoptosis. Additionally, the overexpression of *LcβLectin* in macrophages modulates key immune signaling pathways, enhancing the fish’s innate immune response. These findings highlight *LcβLectin*’s potential as a novel disease resistance gene in aquaculture and provide a foundation for developing strategies to improve viral disease resistance in fish.

## Figures and Tables

**Table 1:** Identity analysis of the amino acid sequences of LcβLectin protein in *Larimichthys crocea* and other species.

Latin Name	Foreign Name	Genbank Accession	Identity (%)
*Larimichthys crocea*	Large yellow croaker	XP_010736086.1	100.00
*Nibea albiflora*	Yellow drum	WEU52361.1	90.37
*Echeneis naucrates*	Live sharksucker	XP_029364677.1	84.44
*Gasterosteus aculeatus*	Three-spined stickleback	XP_040046198.1	82.96
*Pungitius pungitius*	Ninespine stickleback	XP_037331494.1	82.22
*Oplegnathus fasciatus*	Rock bream	ADV35589.1	81.48
*Homo sapiens*	Human	NP_002296.1	40.46
*Equus caballus*	Horse	AQX42328.1	40.00
*Mus musculus*	Mouse	AAH99479.1	39.23
*Xenopus laevis*	Frog	AAK11514.1	39.20
*Gallus gallus*	Chicken	NP_990826.1	39.06
*Centrocercus urophasianus*	Greater sage-grouse	XP_042693815.1	39.06
*Bombina bombina*	Fire-bellied toad	XP_053577272.1	38.71

**Table 2:** Quality and mapping statistics for reads from each library.

Group	Raw Reads (M)	Clean Reads (M)	Clean Bases (Gb)	Q30 (%)	Uniquely Mapping Gene Ratio (%)
c1	41.37	40.78	6.14	96.98	94.67
c2	51.29	50.55	7.60	97.05	94.59
c3	43.54	42.96	6.46	97.12	94.60
LcLec-1	53.64	52.90	7.95	97.13	94.10
LcLec-2	58.58	57.81	8.70	97.16	94.32
LcLec-3	70.95	70.01	10.54	97.73	94.34

## Data Availability

The data supporting the findings of this study are available within the article. Additional datasets generated or analyzed during the current study are available from the corresponding author upon reasonable request. RNA sequencing data were deposited in NCBI Sequence Read Archive (SRA) under accession number PRJNA1188791.

## Supplementary Materials

The following supporting information can be downloaded at: https://www.mdpi.com/article/10.3390/ijms26073251/s1.
