# Supplementary material for: Characterization and Immune Functions of LcβLectin from Large Yellow Croaker (Larimichthys crocea): A Potential Antiviral Defense Molecule
Source: Int J Mol Sci. 2025 Mar 31;26(7):3251. doi: 10.3390/ijms26073251 (PMC11989850; doi:10.3390/ijms26073251)
Supplement: Supplementary file 1 [file ijms-26-03251-s001.zip › ijms-3517025-supplementary.pdf]

Supplementary Table S1. Primers used in this study.

Primers	Sequence (5’-3’)	Purpose
LcβLectin-F	CCCTGGGATCCCCGGAATTCATGGTGAATGGTATGATCGTAAAGAA	ORF amplification
LcβLectin-R	CACGATGCGGCCGCTCGAGTTACTTGATCTCAAAGCTTCTGACACG	
LcβLectin-qF	GGGGAGGAATTCAAGATCGTCAT	RT-qPCR
LcβLectin-qR	CCATCGAAGCTGACAACGGA	
β-actin-qF	TTATGAAGGCTATGCCCTGCC	
β-actin-qR	TGAAGGAGTAGCCACGCTCTGT	
rsad2-qF	AGTGTCAGCATCGTCAGCAA	
rsad2-qR	TGGCACCAGTTGCGAATCTT	
trim25-qF	AGCGAGGTTATTCTGACCGC	
trim25-qR	TTCGGATTTTCCCGGTGAGG	
irf3-qF	AAACCTCTGCTCATCCCGTG	
irf3-qR	ATCCAAGAACTGCTCCGCAA	
irf7-qF	CACCTCTGCAGCCAACCATA	
irf7-qR	TTGAGCTCAGGGACCTCGTA	
casp3-qF	GCCTCGTTCGTCTGTGTTCT	
casp3-qR	AGATCAGTGCCTCGACAAGC	
casp7-qF	CTCGGTTAAAGCTGCGGAGA	
casp7-qF	GCCGGTTCTCCAGACATACT	
mdm2-qF	GCGTCGAGTTCGAGGTAGAG	
mdm2-qF	CTCGTACACCTGATCGTCCG	
*LcβLectin-s*F	CTACCGGACTCAGATCTCGAGATGGTGAATGGTATGATCGTAAAGAA	subcellular

*LcβLectin-s*R	ACCGTCGACTGCAGAATTCGTTACTTGATCTCAAAGCTTCTGACACG	localization
*LcβLectin-o*F	AACGGGCCCTCTAGACTCGAGATGGTGAATGGTATGATCGTAAAGAA	overexpression
*LcβLectin-o*R	AGTCCAGTGTGGTGGAATTCTTACTTGATCTCAAAGCTTCTGACACG	

Note: *Eco*R I（GAATTC）and *Xho* I（CTCGAG）enzyme restriction sites are underlined.
